# Piceatannol Attenuates Renal Fibrosis Induced by Unilateral Ureteral Obstruction via Downregulation of Histone Deacetylase 4/5 or p38-MAPK Signaling

**DOI:** 10.1371/journal.pone.0167340

**Published:** 2016-11-30

**Authors:** Sin Young Choi, Zhe Hao Piao, Li Jin, Jung Ha Kim, Gwi Ran Kim, Yuhee Ryu, Ming Quan Lin, Hyung-Seok Kim, Hae Jin Kee, Myung Ho Jeong

**Affiliations:** 1 Heart Research Center of Chonnam National University Hospital, Gwangju, Republic of Korea; 2 The Second Hospital of Jilin University, Changchun, China; 3 Jilin Hospital Affiliated with Jilin University, Jilin, China; 4 Yanbian University Hospital, Jilin Yanbian, China; 5 Department of Forensic Medicine, Chonnam National University Medical School, Gwangju, Republic of Korea; University of Louisville, UNITED STATES

## Abstract

Piceatannol, a resveratrol metabolite, is a phenolic compound found in red wine and grapes. We investigated the effect of piceatannol on renal fibrosis and histone deacetylase (HDAC) expression in a mouse model of unilateral ureteral obstruction (UUO). Fibrosis was established by UUO and piceatannol was intraperitoneally injected for 2 weeks. Piceatannol suppressed extracellular matrix (ECM) protein deposition including collagen type I and fibronectin as well as connective tissue growth factor (CTGF) and α-smooth muscle actin (α-SMA) in UUO kidneys. However, the expressions of epithelial-mesenchymal transition (EMT) marker genes, such as N-cadherin and E-cadherin, were not changed in the kidneys after UUO. Masson’s trichrome staining and fluorescence immunostaining showed that piceatannol administration attenuated collagen deposition in UUO kidneys. HDAC1, HDAC4, HDAC5, HDAC6, and HDAC10 protein expression was upregulated in UUO kidneys, whereas that of HDAC8 was downregulated. Piceatannol treatment significantly reduced HDAC4 and HDAC5 protein expression. Further, piceatannol attenuated phosphorylation of p38 mitogen-activated protein kinase (p38-MAPK) in UUO kidneys, but not that of transforming growth factor beta1-Smad2/3. These results suggest that class I HDACs and class IIa/b HDACs are involved in renal fibrosis development. Piceatannol may be a beneficial therapeutic agent for treating renal fibrosis via reduction of HDAC4 and HDAC5 protein expression or suppression of the p38-MAPK signaling pathway.

## Introduction

Renal fibrosis is characterized by the accumulation of extracellular matrix (ECM) proteins, activation of myofibroblasts and fibroblasts, and tubular atrophy [1–3]. During fibrosis, interstitial fibroblast activation, pericyte differentiation, epithelial-mesenchymal transition (EMT) of tubular epithelial cells, and recruitment of fibrocytes are involved in the activation of myofibroblasts [4,5]. Unilateral ureteral obstruction (UUO) is a representative model of renal fibrosis and can be used for the evaluation of therapeutic agents for renal diseases [6,7].

Imbalance of histone deacetylase (HDAC) expression or activity is implicated in several diseases. HDACs are divided into four HDAC classes: class I HDACs (HDAC1, HDAC2, HDAC3, and HDAC8); class IIa HDACs (HDAC4, HDAC5, HDAC7, and HDAC9); class IIb HDACs (HDAC6 and HDAC10); class III (Sirt1-7); and class IV (HDAC11). HDAC inhibitors are effective in cancer, cardiac hypertrophy, and inflammation [8–10]. Furthermore, HDAC inhibitors suppress fibrosis in organs such as the heart and kidneys [11,12] as shown *in vivo* as well as *in vitro*, suggesting that HDACs may be therapeutic targets for treating fibrosis. For example, class I HDACs are activated in transforming growth factor β1- (TGF-β1) treated kidney epithelial cells [13] and are involved in the development of EMT and the ECM in fibrosis with [14] or without [15] diabetes. HDAC6, a class IIb HDAC, may be a target for hypertension-induced kidney fibrosis [16]. Class IIa HDACs (HDAC4/5/7) are related to diabetes-induced fibrosis [17].

Piceatannol, a natural polyphenolic stilbene compound, is a metabolite of resveratrol found in red wine. Piceatannol shows several biological activities, including anticancer, anti-inflammatory, anti-oxidative, anti-allergic, anti-adipogenesis, and anti-hypertrophic effects [18–25]. Piceatannol may potentially be protective against cardiovascular diseases, allergy, cancer, and inflammatory diseases. However, only few studies have shown that piceatannol plays a beneficial role in kidney diseases. For example, one study showed that piceatannol in combination with low doses of cyclosporine A prevented kidney allograft rejection [26]. More recently, another study showed a mild renoprotective effect of piceatannol in obese Zucker rats [27]. However, the effect of piceatannol on renal fibrosis and the underlying regulatory mechanism have not been fully investigated.

Resveratrol is a compound very similar to piceatannol. There are several publications related to resveratrol and renal fibrosis. For example, resveratrol inhibits renal interstitial fibrosis through a variety of mechanisms, including the regulation of AMP-activated protein kinase (AMPK)/NAPDH oxidase 4 (NOX4)/reactive oxygen species (ROS) pathway [28], downregulation of nuclear factor-κB (NF-κB) [29], or regulation of the transforming growth factor β (TGF-β) pathway [30].

Considering the protective effect of resveratrol in renal diseases, we hypothesize that piceatannol may have a beneficial effect on renal fibrosis. In this study, we investigated the effect of piceatannol on renal fibrosis in a mouse model of UUO. Furthermore, we assessed the relevance of HDAC expression and the TGF-β1-induced Smad-dependent or Smad-independent signaling pathway in the anti-fibrotic effect of piceatannol.

## Materials and Methods

### Animal Experiments

C57BL/6 male mice (7-week-old) weighing 20~22 g were purchased from ORIENT BIO (Gyeonggi-do, South Korea). All animal experiments were approved by the Animal Experiment Committee of the Chonnam National University Medical School (CNU IACUC-H-2015-52) and were carried out in accordance with the Guide for the Care and Use of Laboratory Animals (US National Institutes of Health Publication, 8^th^ edition, 2011). UUO was performed as follows. After anesthesia induction by using an intraperitoneal injection of ketamine (70 mg/kg) and xylazine (14 mg/kg), a midline incision was made to expose the abdominal cavity and the left ureter was ligated with 6–0 silk. The contralateral/right kidney served as a control. One day after surgery, piceatannol (50 mg/kg/day) or vehicle (1.5% DMSO in 0.9% saline/day) was intraperitoneally injected 10 times during 2 weeks.

### Materials and Antibodies

Piceatannol was purchased from Future Chem (Seoul, Korea). Anti-alpha smooth muscle actin (α-SMA; 1:1000, sc-130617), anti-CTGF (1:1000, sc-14939), anti-HDAC3 (1:1000, sc-11417), anti-HDAC4 (1:1000, sc-11418), anti-HDAC5 (1:1000, sc-133225), anti-TGF-β1 (1:1000, sc-146), anti-JNK (1:1000, sc-7345), anti-ERK1 (1:1000, sc-271269), and anti-GAPDH (1:1000, sc-32233) antibodies were purchased from Santa Cruz Biotechnology (Dallas, TX, USA). Primary antibodies against collagen type I (1:1000, ab34710), HDAC2 (1:1000, ab12169), HDAC8 (1:1000, ab137474), and HDAC10 (1:1000, ab53096) were purchased from Abcam (Cambridge, MA, USA). Anti-fibronectin antibody (1:1000, MA5-11981) was purchased from Thermo Fisher Scientific (Waltham, MA, USA). Anti-HDAC1 antibody (1:1000, 06–720) was purchased from Merck Millipore (Darmstadt, Germany). Anti-HDAC6 (1:1000, 7612), anti-Smad3 (1:1000, 9523), anti-Smad2 (1:1000, 3103), anti-Smad4 (1:1000, 9515), anti-p-Smad3 (1:1000, 9520), anti-p-JNK (1:1000, 9251), anti-p-p38 (1:1000, 4511), anti-p-ERK1/2 (1:1000, 4370), and anti-p38 (1:1000, 8690) antibodies were purchased from Cell Signaling Technology (Danvers, MA, USA).

### Western Blot Analysis

Western blotting was performed as described previously [31]. Cell lysates were prepared with RIPA buffer (150 mM NaCl, 1% Triton X-100, 1% sodium deoxycholate, 50 mM Tris-HCl pH 7.5, 2 mM EDTA, 1 mM PMSF, 1 mM DTT, 1 mM Na_3_VO_4_, and 5 mM NaF) containing a protease inhibitor cocktail (Calbiochem, EMD Millipore Corp., Billerica, MA, USA). Proteins were separated using 8% SDS-PAGE and were then transferred to polyvinylidene difluoride (PVDF) membranes. The membranes were probed with the indicated antibodies and developed using Immobilon Western Detection Reagents (Millipore, Billerica, MA, USA). The Bio-ID software was used to quantify protein expression (Vilber Lourmat, Eberhardzell, Germany). All experiments were performed in triplicate.

### Quantitative Real Time Polymerase Chain Reaction

Total RNA was isolated with TriZol reagent (Invitrogen Life Technologies, Waltham, MA, USA), and 1 μg of RNA was used for the reverse transcription reaction using TOPscript RT DryMIX (Enzynomics, Daejeon, South Korea). The mRNA amounts were determined using the SYBR Green PCR kit (Enzynomics, Daejeon, South Korea). The relative expression level of the indicated genes was compared to that of GAPDH by using the 2-^ΔΔct^ method. PCR was performed using the following oligonucleotide primers: for collagen type I, sense, 5’-GAGCGGAGAGTACTGGATCG-3’, and antisense, 5’-GCTTCTTTTCCTTGGGGTTC-3’; for fibronectin, sense, 5’- GATGCACCGATTGTCAACAG-3’, and antisense, 5’-TGATCAGCATGGACCACTTC-3’; for CTGF, sense, 5’-CAAAGCAGCTGCAAATACCA-3’, and antisense, 5’- GGCCAAATGTGTCTTCCAGT-3’; for α-SMA, sense, 5’-ACTGGGACGACATGGAAAAG-3’, and antisense, 5’-AGAGGCATAGAGGGACAGCA-3’; for GAPDH, sense, 5’- GCATGGCCTTCCGTGTTCCT-3’, and antisense, 5’- CCCTGTTGCTGTAGCCGTATTCAT-3’.

### Histology and Masson’s Trichrome Staining

Kidney tissues were fixed with 4% paraformaldehyde, embedded in paraffin, and cut into 3-μm-thick sections. Hematoxylin and eosin (H&E) staining was performed to assess the histological morphology. The kidney tissue section slides were incubated in Gill’s hematoxylin for 5 min, washed with tap water, incubated in 95% ethanol, and stained with eosin and phloxine for 1 min. Subsequently, the sections were dehydrated in ethanol and xylene, and were mounted with Canada balsam.

For Masson’s trichrome staining, after deparaffinization with xylene, the sections were treated with Bouin’s solution at 56°C for 30 min and were washed under running tap water until the sections were clear. The sections were subsequently stained with Weigert’s hematoxylin (A:B = 1:1), followed by staining with Biebrich Scarlet/Acid Fuchsin solution for 10 min and washing with distilled water. The sections were incubated with phosphotungstic acid/phosphomolybdic acid solution for 10 min and were treated with Aniline Blue solution for 15 min. They were subsequently incubated with acetic acid for 1 min and were dehydrated with ethanol and xylene. Collagen depositions, nuclei, and muscle fibers were stained blue, black, and red, respectively.

### Immunofluorescence Staining

After standard histological processing procedures, immunostaining was performed using collagen type I antibody. The sections were deparaffinized in xylene and rehydrated with graded alcohol. Antigen retrieval was performed using 10 mM citrate-phosphate buffer (pH 6.0), the sections were subsequently blocked with 3% goat serum for 1 h, incubated with anti-collagen type I antibody (1:100, Abcam) at 4°C overnight, and incubated with Alexa Fluor 488 goat anti-rabbit IgG antibody (1:200, Invitrogen) for 1 h. Sudan black B solution was used to reduce autofluorescence. The sections were counterstained with DAPI and images were acquired using fluorescence microscopy (Nikon Eclipse 80*i*, Tokyo, Japan).

### Statistics

Statistical analysis was performed using one-way ANOVA followed by the Bonferroni post-hoc test for comparative analysis between the treatment groups (GraphPad Prism, version 5.0; GraphPad Software, La Jolla, CA, USA). The data are presented as the means ± SD. A P value of <0.05 was considered statistically significant.

## Results

### Piceatannol reduces ECM protein and fibrosis marker expression in the UUO kidney

**Fig 1:**
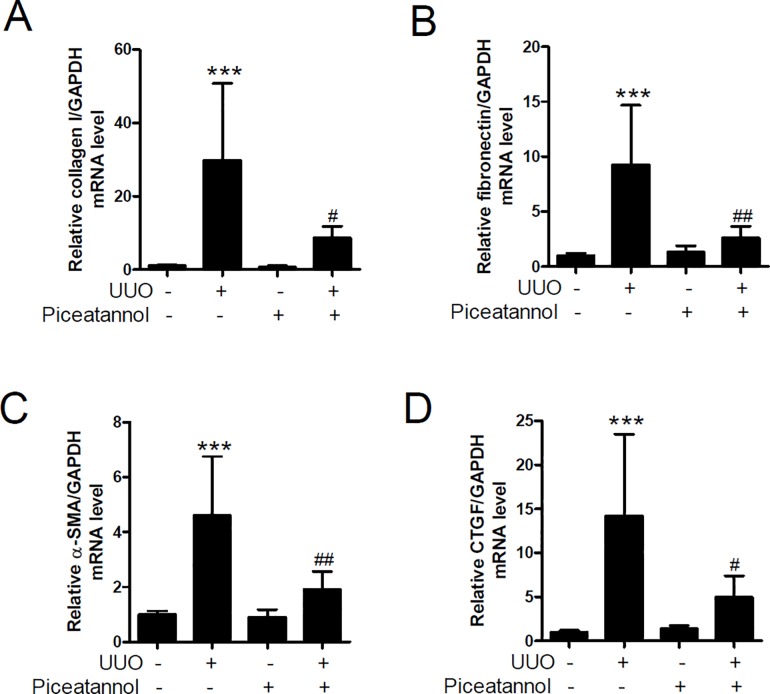
Piceatannol reduces the mRNA level of fibrosis-related genes in the UUO kidney. (A-D) One day after UUO surgery, the mice received an intraperitoneal injection of vehicle or piceatannol (50 mg/kg/day) for 2 weeks. The transcription level of collagen type I (collagen type I), fibronectin, alpha smooth muscle actin (α-SMA), and connective tissue growth factor (CTGF) was determined using qRT-PCR in kidney tissues from UUO mice treated with vehicle or piceatannol. The data are expressed as the means ± SD of the mice (n = 6 per group). ***P<0.001 compared with the contralateral kidney. ^#^*P*<0.05 and ^##^*P*<0.01 compared with the UUO kidney.

To determine whether piceatannol might have a therapeutic effect on renal fibrosis, we administrated vehicle or piceatannol (50 mg/kg/day) to UUO mice for 2 weeks. To evaluate whether piceatannol could affect the transcript levels of ECM and fibrosis markers, we performed qRT-PCR in kidney tissues. The mRNA levels of collagen type I, fibronectin, CTGF, and α-SMA were significantly enhanced in the UUO group compared with those in the control group. The increase was inhibited by piceatannol (Fig 1A–1D).

**Fig 2:**
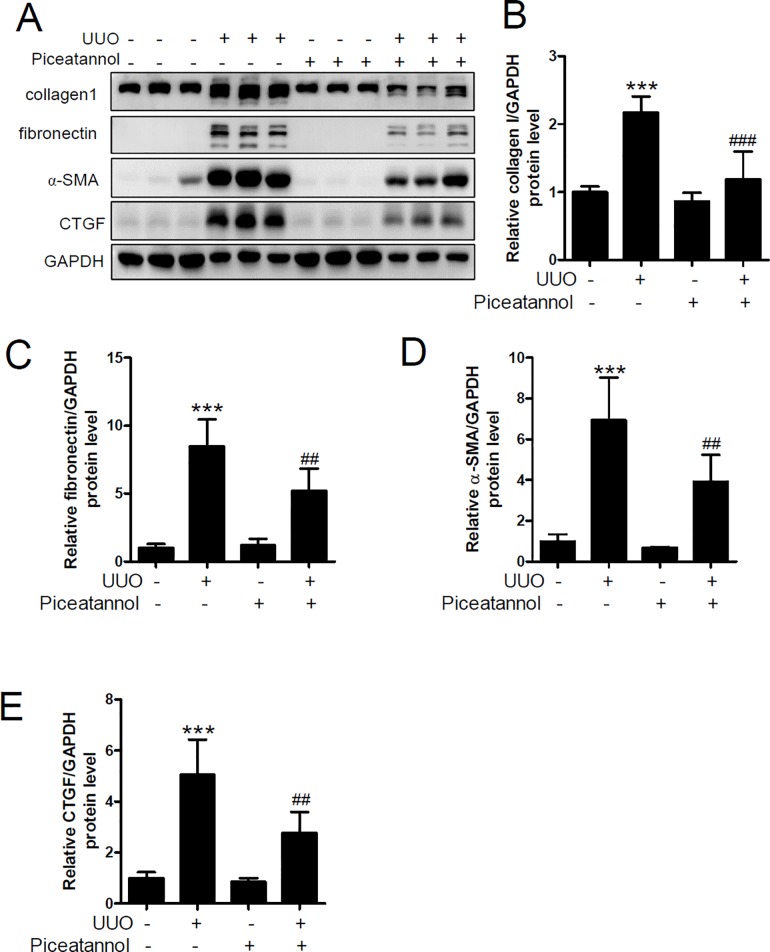
Piceatannol reduces extracellular matrix (ECM) protein and fibrosis marker protein expression in the UUO kidney. One day after UUO surgery, the mice received an intraperitoneal injection of vehicle or piceatannol (50 mg/kg/day) for 2 weeks. (A) Protein lysates from kidney tissues were prepared and were analyzed using western blotting. Anti-collagen 1, fibronectin, α-SMA, and CTGF antibodies were used. GADPH was used as a loading control. Representative immunoblots. (B-E) Protein levels were quantified using densitometry. The data are expressed as the means ± SD of the mice (n = 6 per group). ***P<0.001 compared with the contralateral kidney. ^##^*P*<0.01 and ^###^*P*<0.001 compared with the UUO kidney.

As shown in Fig 2A–2C, UUO increased the ECM proteins collagen type I and fibronectin, but the increase was significantly suppressed by piceatannol treatment. α-SMA is a myofibroblast marker involved in organ fibrosis [32]. We observed that UUO-induced α-SMA expression was significantly reduced by piceatannol administration (Fig 2A and 2D). CTGF is a matricellular protein related to tissue and wound repair and fibrotic pathology [33]. UUO-induced CTGF expression was ameliorated by treatment with piceatannol (Fig 2A and 2E). No changes in the expression of ECM proteins and fibrosis markers were observed in the control mice treated with piceatannol.

### EMT gene expression is not changed in the UUO kidney

**Fig 3:**
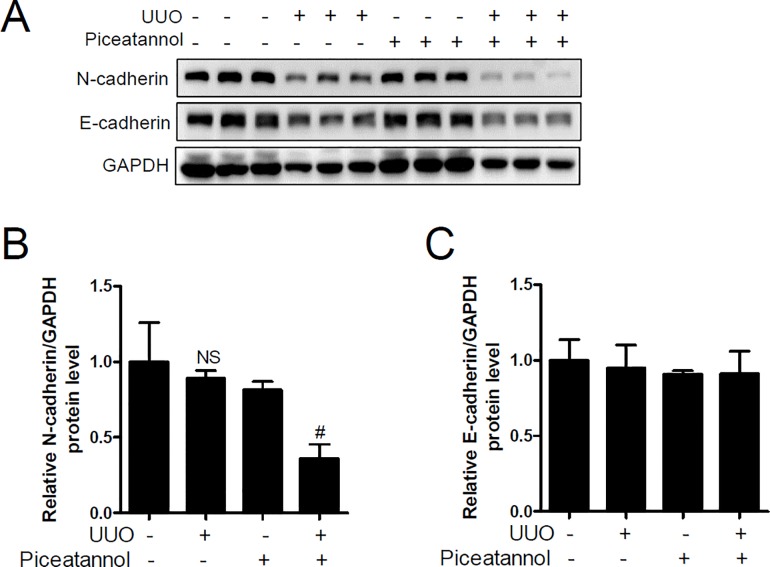
Epithelial-mesenchymal transition (EMT) gene expression is not observed in the UUO kidney. One day after UUO surgery, the mice received an intraperitoneal injection of vehicle or piceatannol (50 mg/kg/day) for 2 weeks. (A) Protein lysates from kidney tissues were prepared and were analyzed using western blotting. N-cadherin and E-cadherin antibodies were used. GADPH was used as a loading control. Representative immunoblots. (B-C) Protein levels were quantified using densitometry. The data are expressed as the means ± SD of the mice (n = 6 per group). NS indicates not significant. ^#^*P*<0.05 compared with the UUO kidney.

EMT is characterized by the loss of epithelial cell polarity and cell-to-cell adhesion as well as gain of mesenchymal cell migration. EMT is associated with renal fibrosis [34,35]. We performed western blot analysis to investigate whether piceatannol could affect EMT. Unexpectedly, we found that N-cadherin protein expression was not increased in UUO kidneys but that piceatannol significantly reduced N-cadherin expression (Fig 3A and 3B). In addition, we observed that E-cadherin was not downregulated in UUO kidneys (Fig 3A and 3C). Thus, these results indicated that downregulation of E-cadherin and upregulation of N-cadherin was not implicated in UUO-induced renal fibrosis.

### Piceatannol does not affect UUO-induced tubular atrophy

**Fig 4:**
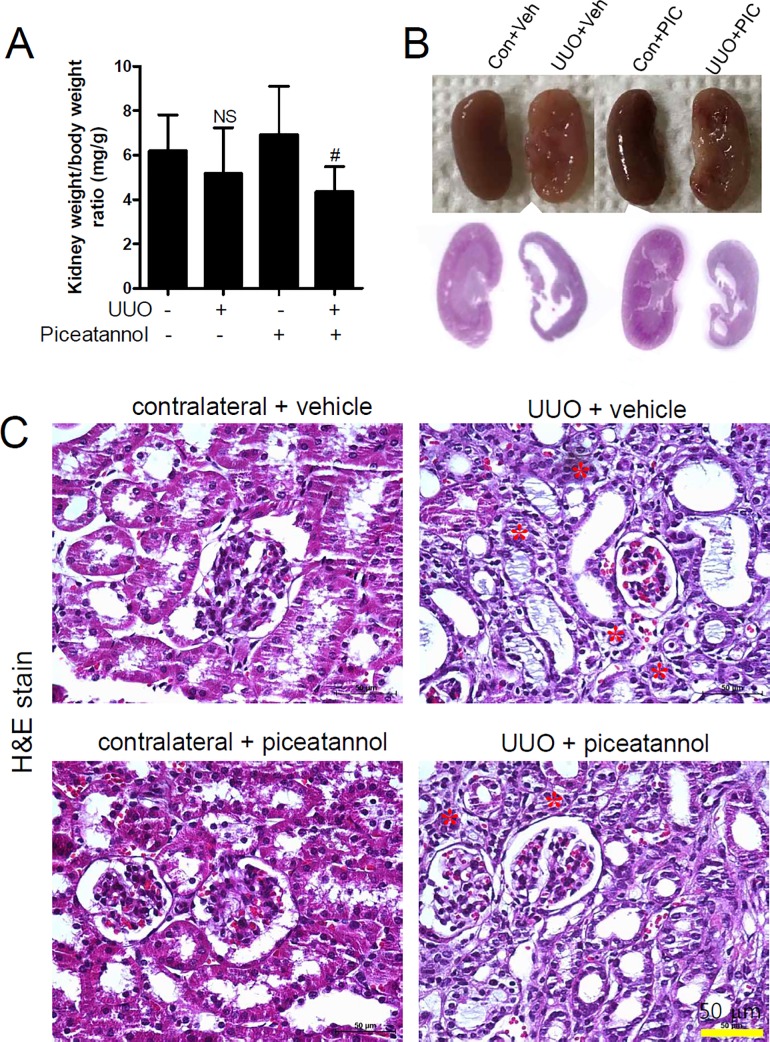
Piceatannol does not affect UUO-induced tubular atrophy. (A-C) One day after UUO surgery, the mice received an intraperitoneal injection of vehicle or piceatannol (50 mg/kg/day) for 2 weeks. (A) The kidney weight to body weight ratio (mg/g) is shown. (B) Representative photomicrographs of the kidneys are shown (upper panel). Left panel is organized as follows: contralateral + vehicle (Con + Veh), UUO + vehicle (UUO + Veh), contralateral + piceatannol (Con + PIC), and UUO + piceatannol (UUO + PIC). Representative images of hematoxylin & eosin (H&E) staining are shown at a lower magnification (lower panel). (C) H&E staining was performed to examine changes in the renal structure: Con + Veh, Con + PIC, UUO + Veh, and UUO + PIC. Scale bar is 50 μm. Asterisks (*, red) indicates tubular atrophy.

We examined the changes in kidney weight in UUO. A tendency for a decreased weight of the UUO kidney was observed compared to that of the contralateral kidney, regardless of treatment with piceatannol (Fig 4A).

Severe morphological changes or a rumpled appearance were observed in the UUO kidney compared to the contralateral kidney (Fig 4B, upper panel). Histologically, the contralateral kidney had a normal cortex, outer medulla, and inner medulla structure, whereas the medulla structure of the UUO kidney was incomplete (Fig 4B, lower panel). Tubular atrophy and dilatation (Fig 4C, top right panel) were observed in the UUO kidney relative to the vehicle-treated contralateral kidney (Fig 4C, top left panel). However, these changes were not attenuated by piceatannol treatment (Fig 4C, bottom right panel).

### Piceatannol ameliorates renal fibrosis in the UUO kidney

**Fig 5:**
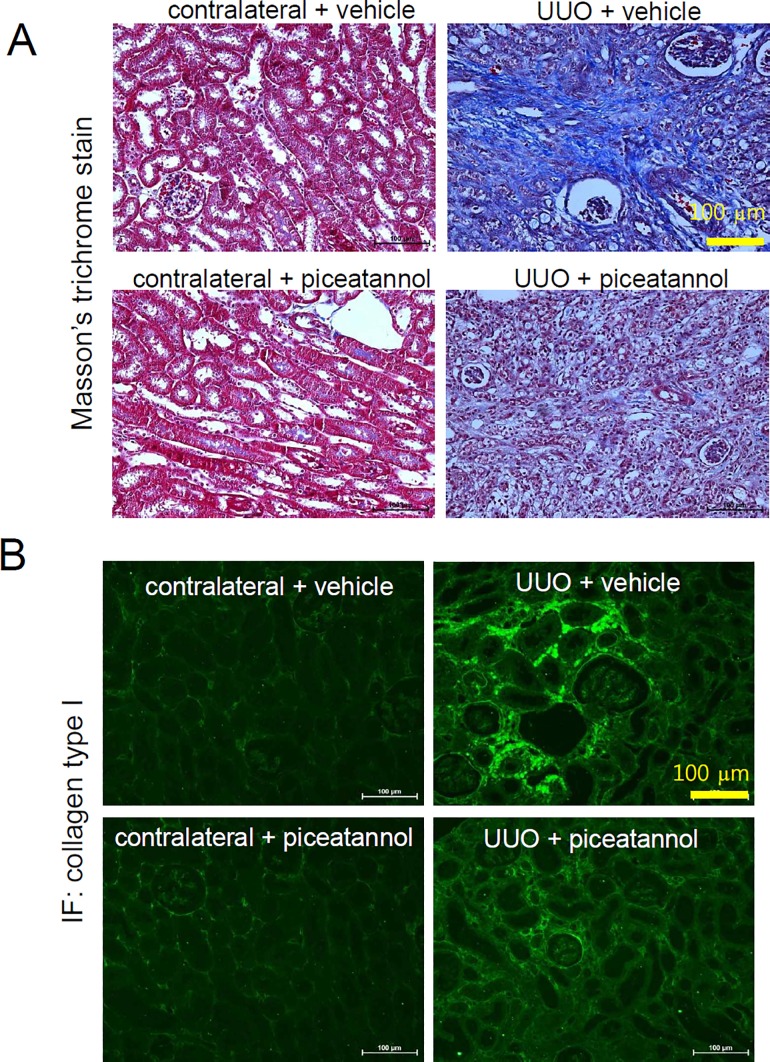
Piceatannol decreases renal fibrosis in the UUO kidney. (A-B) One day after UUO surgery, mice were received an intraperitoneal injection of vehicle or piceatannol (50 mg/kg/day) for 2 weeks. (A) Masson’s trichrome staining was performed to examine interstitial fibrosis: contralateral + vehicle, contralateral + piceatannol, UUO + vehicle, and UUO + piceatannol. Scale bar is 100 μm. (B) Immunofluorescent staining using anti-collagen type I antibody. A representative photomicrograph is shown. Scale bar is 100 μm.

We performed Masson’s trichrome staining to further assess whether piceatannol might be a therapeutic agent for renal fibrosis. As shown in Fig 5A, deposition of interstitial collagen was not observed in the vehicle-treated contralateral kidney and piceatannol-treated contralateral kidney (left, upper panel and lower panel). Interstitial fibrosis was increased in the UUO kidney (right, upper panel), whereas it was attenuated by piceatannol treatment (right, lower panel). Immunofluorescence staining showed that collagen type I expression was increased in the peritubular and periglomerular interstitium in the UUO kidney, which was attenuated by piceatannol treatment (Fig 5B).

### HDAC1 protein expression is upregulated in the UUO kidney

Dysregulation of HDACs is associated with several diseases [36]. We performed western blot analysis to investigate whether HDAC expression was changed in the UUO kidney.

**Fig 6:**
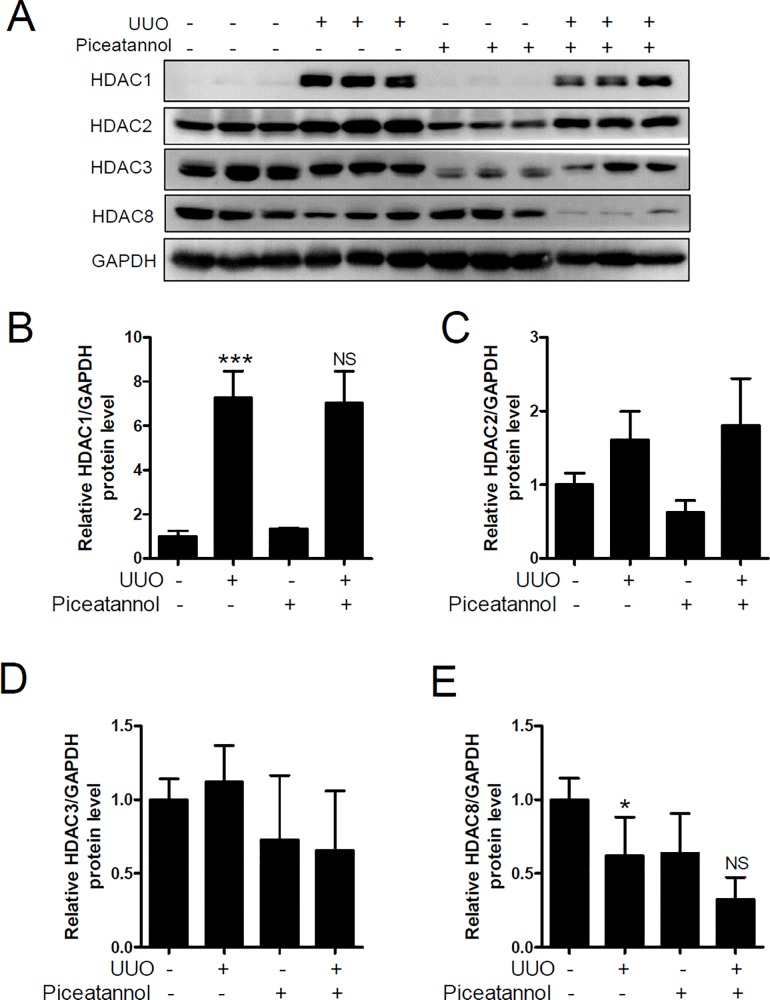
HDAC1 protein expression is upregulated in the UUO kidney. (A) Kidney protein lysates were analyzed using western blotting. Antibodies against HDAC1, HDAC2, HDAC3, and HDAC8 were used. GAPDH was used as a loading control. (B-E) Quantification analysis was performed using densitometry. The data are expressed as the means ± SD of the mice (n = 6 per group). *P<0.05 and ***P<0.001 compared with the contralateral kidney. NS indicates not significant.

First, we examined the protein expression of class I HDACs including HDAC1, HDAC2, HDAC3, and HDAC8. As shown in Fig 6A, HDAC1 protein levels were significantly increased in the UUO kidney than that in the contralateral kidney. However, piceatannol treatment did not reduce the level of HDAC1 protein (Fig 6B). HDAC2 and HDAC3 protein expression was unchanged in the UUO kidney (Fig 6C and 6D), whereas that of HDAC8 was decreased in the UUO kidney (Fig 6E). These results indicate that class I HDACs may not play a major role in UUO-induced renal fibrosis.

### Piceatannol attenuates UUO-induced HDAC4 and HDAC5 protein expression

**Fig 7:**
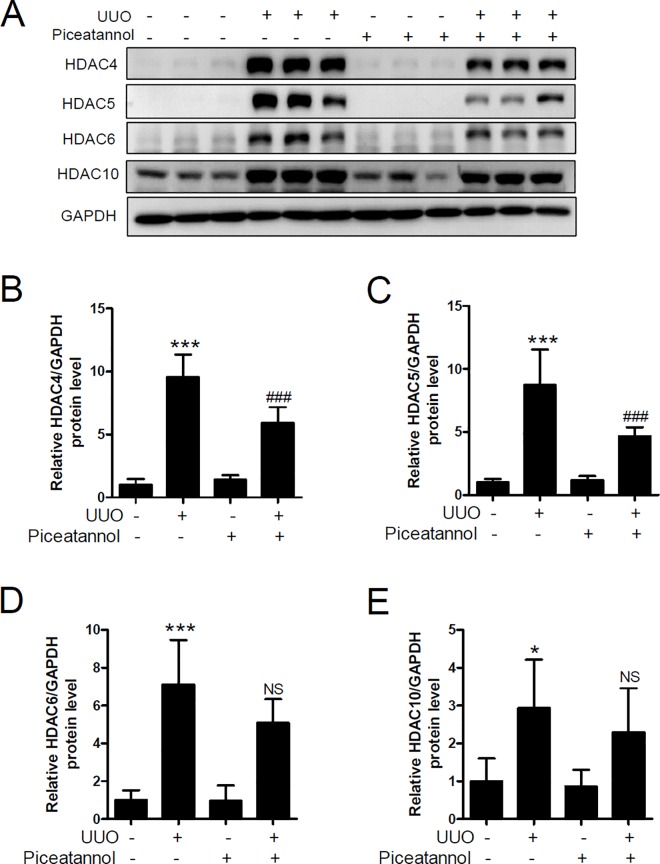
Piceatannol attenuates the expression of UUO-induced renal HDAC5/HDAC6 protein. (A) Kidney lysates were used for western blot analysis. Antibodies against HDAC4, HDAC5, HDAC6, and HDAC10 were used. GAPDH was used as a loading control. (B-E) Quantification analysis was performed using densitometry. The data are expressed as the means ± SD of the mice (n = 6 per group). *P<0.05 and ***P<0.001 compared with the contralateral kidney. ^###^*P* <0.001 compared with the UUO kidney. NS indicates not significant compared with the UUO kidney.

Class II HDACs are divided into two subgroups. Class IIa includes HDAC4, HDAC5, HDAC7, and HDAC9; class IIb includes HDAC6 and HDAC10 [37]. We next examined expression of HDAC4, HDAC5, HDAC6, and HDAC10 in the UUO kidney. As shown in Fig 7A, the protein levels of HDAC4, HDAC5, HDAC6, and HDAC10 were significantly increased in the UUO kidney compared to those in the contralateral kidney. Treatment with piceatannol reduced the UUO-induced protein expression of HDAC4 and HDAC5 (Fig 7B and 7C) but not that of HDAC6 and HDAC10 (Fig 7D and 7E).

### Piceatannol attenuates UUO-induced p38-MAPK activation but not TGF-β1-Smad2/3 pathway activation

**Fig 8:**
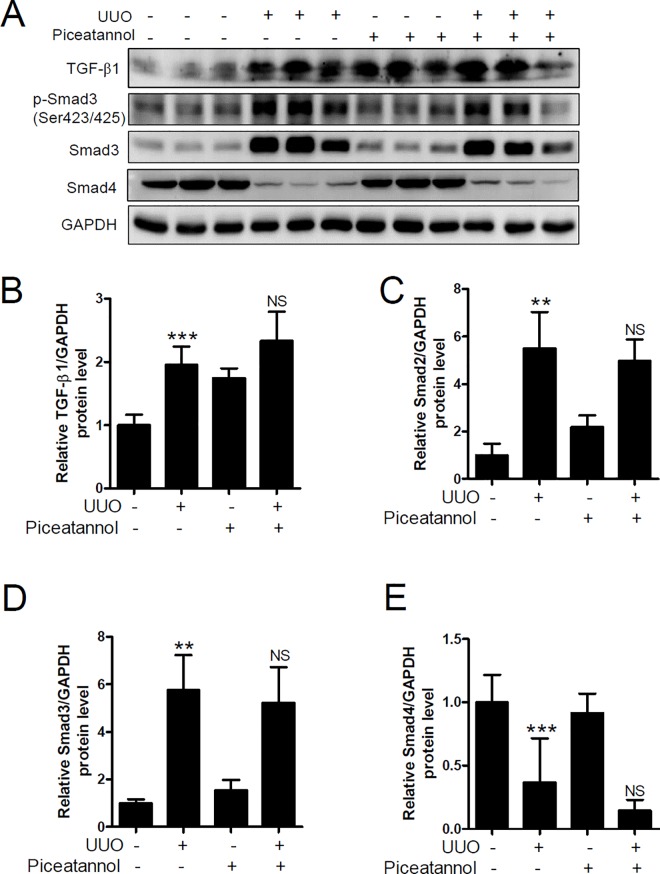
Piceatannol does not suppress TGF-β1-Smad signaling in the UUO kidney. (A) Kidney lysates were used for western blot analysis. Antibodies against TGF-β1, p-Smad3 (Ser423/425), Smad3, Smad2, and Smad4 were used. GAPDH was used as a loading control. (B-E) Quantification analysis was performed using densitometry. The data are expressed as the means ± SD of the mice (n = 6 per group). **P<0.01 and ***P<0.001 compared with the contralateral kidney. NS indicates not significant compared with the UUO kidney.

**Fig 9:**
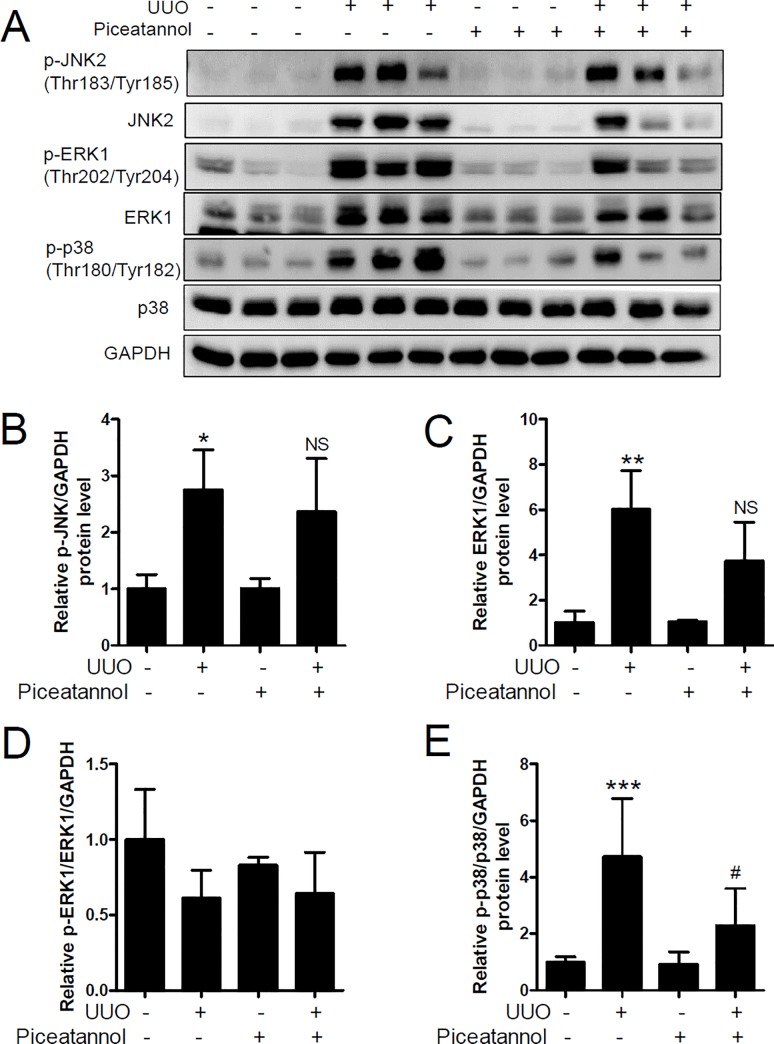
Piceatannol attenuates phosphorylated p38 MAPK expression in the UUO kidney. (A) Kidney lysates were used for western blot analysis. Antibodies against p-JNK2 (Thr183/Tyr185), JNK2, p-ERK1 (Thr202/Tyr204), ERK1, p-p38 (Thr180/Tyr182), and p38 were used. GAPDH was used as a loading control. (B-E) Quantification analysis was performed using densitometry. The data are expressed as the means ± SD of the mice (n = 6 per group). *P<0.05, **P<0.01, and ***P<0.001 compared with the contralateral kidney. ^#^*P* <0.05 compared with the UUO kidney. NS indicates not significant compared with the UUO kidney.

TGF-β/Smad signaling is a critical mediator of renal fibrosis [38]. We performed western blot analysis to determine whether piceatannol affected TGF-β/Smad signaling. Protein expression of TGF-β1, Smad2, and Smad3 was significantly increased in the UUO kidney (Fig 8A–8D), and was not reduced by piceatannol treatment. Similarly, UUO-induced phosphorylation of Smad3 (Ser423/425) was not suppressed by piceatannol treatment. In contrast, Smad4 protein expression significantly reduced in the UUO kidney (Fig 8A and 8E). TGF-β/Smad signaling interacts with MAPK signaling in renal fibrosis [39–42]. To assess whether piceatannol affected TGF-β1-induced MAPK signaling, we examined the protein expression of JNK2, ERK1, and p38 in the UUO kidney. As shown in Fig 9A–9E, the phosphorylated state of JNK2 (Thr183/Tyr185), ERK1 (Thr202/Tyr204), and p38 (Thr180/Tyr182) was increased in the UUO kidney compared to that in the contralateral kidney. Expression of phosphorylated JNK2 and ERK1 proteins was not reduced by piceatannol treatment. Unexpectedly, we observed that the expression of non-phosphorylated JNK2 and ERK1, except p38-MAPK, also increased in the UUO kidney compared to that in the control kidney (Fig 9A). Of note, the ratio of phosphorylated p38 to total p38 significantly reduced in the piceatannol-treated UUO kidney compared to that in the UUO kidney (Fig 9A and 9F).

## Discussion

**Fig 10:**
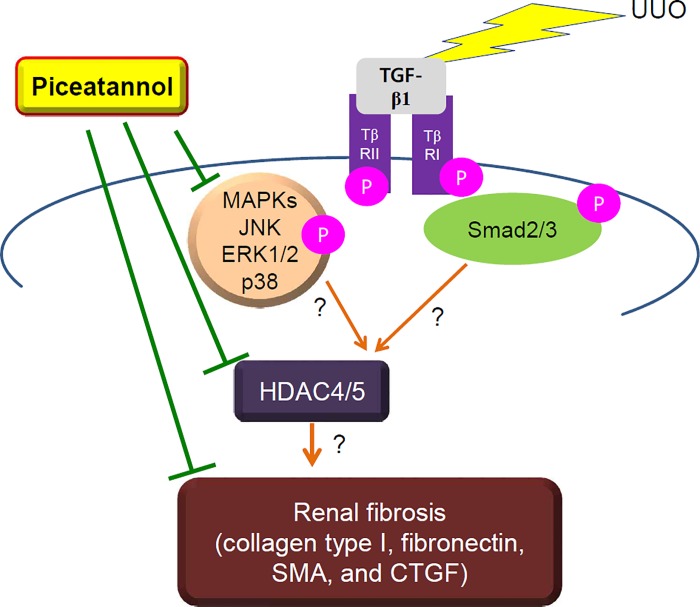
Piceatannol suppresses renal fibrosis by downregulation of the p38-MAPK/HDAC4/5 pathway. Fibrotic stress such as UUO can induce TGF-β1 expression, which causes phosphorylation and upregulation of Smad2/3 or MAPK (JNK2, ERK1/2, or p38) protein expression. It also increases the expression of class II HDACs (HDAC4/5). Piceatannol attenuates the activation of p38-MAPK signaling and the increased HDAC4/5 expression, but not the activation of the TGF-β1/Smad3 signaling pathway. However, whether a direct association between HDAC4/5 and MAPK signaling or between HDAC4/5 and renal fibrosis exists remains unknown. TβRII and TβRI indicate TGFβ receptor II and TGFβ receptor I, respectively.

The present study demonstrates that piceatannol attenuates renal fibrosis in a mouse model of UUO. As explained in Fig 10, we suggest that the anti-fibrotic effect of piceatannol is related to the inhibition of the TGF-β1/Smad-independent pathway but not to that of the TGF-β1/Smad-dependent pathway in the UUO-induced fibrosis model. Unexpectedly, piceatannol treatment did not reduce the upregulation of TGF-β1, Smad2, and Smad3 as well as that of phosphorylated Smad3 expression in the UUO kidney. Of note, piceatannol attenuated phosphorylated p38-MAPK in the UUO kidney. Our findings indicate that the anti-fibrotic effect of piceatannol may be associated with downregulation of class IIb HDAC protein expression (HDAC4/5). However, we could not demonstrate a link between HDAC4/5 and MAPK signaling or TGF-β1/Smad signaling, nor a direct association between HDAC4/5 and renal fibrosis.

UUO kidney tissues exhibit interstitial fibrosis and severe morphological changes. Masson’s trichrome staining showed that the increased accumulation of collagen in the UUO kidney was ameliorated by piceatannol treatment. Piceatannol reduced the collagen type I mRNA and protein level in the obstructed kidney 14 days after UUO surgery. Immunofluorescence staining demonstrated that renal collagen type I expression was decreased by piceatannol treatment. Fibronectin is another ECM glycoprotein involved in cell adhesion, wound healing, and fibrosis [43,44]. In our study, the contralateral kidney showed a low fibronectin expression, whereas the expression was greatly induced in the UUO kidney. Piceatannol treatment significantly suppressed fibronectin mRNA and protein expression in the UUO kidney. Fibroblast-to-myofibroblast transition is implicated in renal fibrosis [45]. α-SMA is a representative marker of myofibroblast activation [46]. We found increased α-SMA mRNA and protein expression in the UUO kidney, and this was reduced by piceatannol treatment. CTGF showed results similar to those observed for α-SMA. The UUO-induced increase in fibrosis-related gene expression including collagen type I, fibronectin, α-SMA, and CTGF was consistent with that found in previous studies [47–49]. The upregulation of fibrosis-related genes is related to changes in renal structure in the UUO kidney. Tubular atrophy and dilatation was observed in the UUO kidney as determined using H&E staining. These morphological changes were consistent with those shown in previous studies [50–52]. N-cadherin is a marker of mesenchymal changes in fibrosis, whereas E-cadherin is a hallmark of epithelial features [35]. In the present study, we did not observe a change in EMT marker gene expression in the UUO kidney.

Studies of the effects of piceatannol on renal fibrosis are limited to obese rat models, while the effects of resveratrol have been studied in several animal models, including models of renal ischemia-reperfusion injury, UUO, diabetes (db/db mice), hypertension (spontaneously hypertensive rats, SHR), renovascular hypertension, and obesity (obese Zucker rats) (Table 1). As shown in Table 1, resveratrol has a protective effect against renal fibrosis and renal injury. Therefore, we expect that piceatannol may have similar renal protective effects as those observed for resveratrol.

**Table 1:** Pharmacological effects of piceatannol and resveratrol on *in vivo* models of renal diseases.

Compound	Effects	Target organ	Animal models	References
Piceatannol	Mild renoprotective effect	Kidney	Obese Zucker rats	Llarena et al., 2016 [27]
Piceatannol	Preventive graft rejection	Kidney	ACI-to-Lewis rats	Fernandez et al., 2002 [26]
Resveratrol	• RenoprotectionReduction of renal interstitial fibrosis • Inhibition of EMT and renal fibrosis	Kidney	UUO rats	• Yang et al., 2016 [29] • Zhang et al., 2016 [67] • Bai et al., 2014 [68]
Resveratrol	Attenuation of renal interstitial fibrosis	Kidney	db/db mice	• He et al., 2016 [28] • Yan et al., 2016 [69]
Resveratrol	Amelioration of renal injury and tubulointerstitial fibrosis	Kidney	SHR rats	Xue et al., 2016 [70]
Resveratrol captopril	Improvement of aortic remodeling and fibrosis	Aorta	2K1C Goldblatt rats	Natalin et al., 2016 [71]
Resveratrol	• Attenuation of renal injury and fibrosis • Protective renal fibrosis	Kidney	• UUO mice or I/R injury mice • UUO mice • UUO mice	• Xiao et al., 2016 [30] • Liang et al., 2014 [72] • Li et al., 2010 [48]
Resveratrol	Protective renal fibrosis	Kidney	5/6^th^ nephrectomized rats	Huang et al., 2014 [73]
Resveratrol	• Attenuation of diabetic nephropathy • Reduction of renal fibrosis	Kidney	Streptozotocin-treated rats	• Wen et al., 2013 [74] • Chen et al., 2011 [75]
Resveratrol	Renoprotection	Kidney	Unilateral nephrectomized rats	Sener at al., 2006 [76]

UUO: unilateral ureteral obstruction

EMT: epithelial-mesenchymal transition

I/R injury: ischemia-reperfusion injury

db/db: spontaneous type 2 diabetic animal model

SHR: spontaneously hypertensive rats

2K1C: two-kidney, one-clip model

HDACs are enzymes that remove the acetyl group at the N-terminal region of histone and non-histone proteins [53]. Dysfunction of HDACs is involved in a variety of diseases [53]. For example, overexpression of HDAC1, HDAC2, HDAC4, HDAC6, and HDAC7 has been observed in various cancers. To the best of our knowledge, the present study is the first to show that HDAC1 and most members of class IIa/b HDACs are associated with renal fibrosis. Among the class I HDACs, HDAC1 expression increased in the UUO kidney, which was not reduced by piceatannol treatment. The observed UUO-induced HDAC1 expression was in accord with the finding of a previous report [15]. Further, Tian et al. reported that HDAC1 mRNA and protein expression increased in aristolochic acid I-induced renal fibrosis [54]. Of the class II HDACs assessed, the protein expression of HDAC4, HDAC5, HDAC6, and HDAC10 highly increased in the UUO kidney. Interestingly, HDAC4 and HDAC5 protein expression was downregulated by piceatannol treatment. Considering the findings that the anti-fibrotic effect of piceatannol was related to the reduced expression of HDAC4 and HDAC5, future studies are needed to determine the effect of class IIa-selective HDAC inhibitors (for example LMK235, a selective HDAC4 and HDAC5 inhibitor) in the UUO model. In addition, it is important to investigate whether the anti-fibrotic effect of piceatannol is related to inhibition of HDAC activity. Pharmacological HDAC inhibitors such as trichostatin A or MS-275 were protective against renal fibrosis in a rodent model of UUO [55–57], indicative of an anti-fibrotic effect of HDAC inhibitors via inhibition of HDAC enzyme activity.

Natural products (piceatannol and resveratrol) are known activators of SIRT1, a class III HDAC. Both these polyphenol compounds increased SIRT1 mRNA and protein expression in the THP-1 monocytic cell line [58]. Piceatannol is also known as a dietary HDAC activator, and binds to a conserved N-terminal domain in SIRT1 [59]. In the present study, we did not examine SIRT1 expression in the UUO kidney. However, a recent study showed that renal fibrosis was inhibited by treatment of UUO mice with the SIRT1/2 inhibitor sirtinol or SIRT1 inhibitor EX527 [60]. This result raises doubts about the protective role of SIRT1 in renal fibrosis.

However, our results showed that administration of piceatannol reduced the UUO-induced class II HDACs (HDAC4 and 5) protein levels. This finding indicates that piceatannol may be considered to be acting a dietary HDAC inhibitor rather than a HDAC activator in this model. As mentioned above, our findings are supported by several studies showing that renal fibrosis was attenuated by the pan-HDAC inhibitor TSA [57], class I HDAC inhibitor MS-275 [56], and HDAC6-selective inhibitor Tubastatin A [16].

Piceatannol is a metabolite of resveratrol and is present in red wine, grapes, berries, and several plants. Sim fruit (*Rhodomyrtus tomentosa*) has a higher piceatannol content than that of blueberries and red grapes [61]. Piceatannol inhibits cancer, inflammation, cardiac hypertrophy, and adipogenesis [24,62,63]. Furthermore, a recent study showed a renoprotective effect of piceatannol by attenuating early-stage nephropathy associated with obesity [64], suggesting the potential efficacy of piceatannol in preventing renal fibrosis.

The area under the plasma concentration curve (AUC) represents the bioavailability of a drug as determined using the plasma drug concentrations. The AUC for piceatannol (8.6 μmol/h/L) was higher than that for resveratrol (4.1 μmol/h/L), suggesting that piceatannol has a higher metabolic stability [65]. A recent report showed enhanced absorption of piceatannol in rats when complexed with α-cyclodextrin [66]. Therefore, we hypothesize that piceatannol can be a useful phytochemical compound to treat or prevent renal fibrosis without causing side-effects.

In summary, our data show that piceatannol can suppress renal fibrosis as well as the expression of fibrosis-related genes in the UUO kidney. The anti-fibrotic effect of piceatannol may be associated with the downregulation of HDAC4 and HDAC5 in kidney fibrosis. Piceatannol inhibits the p38-MAPK signaling pathway, but not the TGF-β/Smad-dependent pathway. Taken together, we suggest that piceatannol can be a potential therapeutic agent in the treatment of renal fibrosis development.

## Abbreviations

CTGF: connective tissue growth factor
ECM: extracellular matrix
EMT: epithelial-mesenchymal transition
ERK1/2: extracellular signal-regulated kinase
HDAC: histone deacetylase
H&E: hematoxylin and eosin
JNK: c-Jun terminal kinase
MAPK: mitogen-activated protein kinase
α-SMA: α-smooth muscle actin
TGF-β1: transforming growth factor beta1
TβRI: TGFβ receptor I
TβRII: TGFβ receptor II
UUO: unilateral ureteral obstruction
qRT-PCR: quantitative real time polymerase chain reaction
